# Painful lower limb nodules as first symptom of resectable pancreatic acinar cell cancer: a case report

**DOI:** 10.1186/s13256-022-03595-1

**Published:** 2022-10-05

**Authors:** S. M. Haenen, J. A. M. G. Tol, S. C. J. van Steen, O. R. Busch, A. Farine Sarasqueta, S. Roshani, A. Wolkerstorfer, M. M. D. van der Linden, J. W. Wilmink, H. C. Post, M. G. Besselink

**Affiliations:** 1 Department of Surgery, Amsterdam UMC, location University of Amsterdam, Amsterdam, The Netherlands; 2 Department of internal medicine, Amsterdam UMC, location University of Amsterdam, Amsterdam, The Netherlands; 3 Department of pathology, Amsterdam UMC, location University of Amsterdam, Amsterdam, The Netherlands; 4 Huid Medisch Centrum, Amsterdam, The Netherlands; 5 Department of medical oncology, Amsterdam UMC, location University of Amsterdam, Amsterdam, The Netherlands; 6 Cancer Center Amsterdam, Amsterdam, The Netherlands

**Keywords:** Pancreatic panniculitis, Acinar cell carcinoma, Cutaneous nodules, Case report

## Abstract

**Background:**

Pancreatic panniculitis is characterized by subcutaneous fat necrosis and is a rare presentation of an underlying pancreatic disease, appearing in approximately 2–3% of all patients with a pancreatic disease. The nodules usually involve the lower extremities. Pancreatic panniculitis is commonly associated with acute or chronic pancreatitis, and occasionally with pancreatic cancer, especially acinar cell carcinoma.

**Case presentation:**

A 77-year-old Caucasian woman with no significant medical history was referred to our center with multiple painful, itchy, and warm red/blue cutaneous nodules on the left lower leg. These skin lesions were consistent with the clinical diagnosis of panniculitis. The skin biopsy obtained showed a predominantly lobular panniculitis with fat necrosis of which the aspect was highly suspicious for pancreatic panniculitis. Further analysis revealed high lipase serum of > 3000 U/L (normal range < 60 U/L), and on computed tomography scan a mass located between the stomach and the left pancreas was seen. Endoscopic ultrasonography-guided fine-needle biopsy confirmed the diagnosis of acinar cell carcinoma. After discussing the patient in the pancreatobiliary multidisciplinary team meeting, laparoscopic distal pancreatectomy including splenectomy and en bloc wedge resection of the stomach due to tumor in-growth was performed. The cutaneous nodules on both legs disappeared 1–2 days after surgery. No long-term complications were reported during follow-up. One year after surgery, the patient presented with similar symptoms as preoperatively. Computed tomography scan showed local recurrence and distal metastases, which were subsequently confirmed by biopsy. She started with palliative folinic acid–fluorouracil–irinotecan–oxaliplatin chemotherapy but stopped after two cycles because of disease progression. The patient died 2 months later, 13 months after surgical resection.

**Conclusion:**

This case illustrates the importance of clinically recognizing cutaneous nodules and pathological recognizing the specific microscopic changes as sign of a (malignant) pancreatic disease.

## Background

Pancreatic panniculitis, first described by Chiari in 1883 [1], is a rare skin manifestation characterized by a specific type of subcutaneous fat necrosis, namely saponification of fatty tissue. It is a rare presentation of a pancreatic disease, appearing in approximately 2–3% of all patients with a pancreatic disease [2]. The disease is commonly associated with acute or chronic pancreatitis, although more rarely it is also associated with pancreatic cancer, especially acinar cell carcinoma [2, 3]. Patients with pancreatic panniculitis most commonly present with erythematous cutaneous nodules on the lower extremities as first manifestation of the underlying pancreatic disease, but nodules at other sites have been reported [4].

The pathophysiology is unclear, but it is believed that the release of pancreatic enzymes, in particular lipase, into the circulating system, leads to this characteristic form of fat necrosis [5, 6].

We describe a case that highlights the importance of recognizing cutaneous nodules as a potential warning sign of pancreatic acinar cell carcinoma.

## Case presentation

A 77-year-old Caucasian women was referred to our dermatology department with multiple painful, itchy, and warm red/blue cutaneous nodules on the left lower leg. The cutaneous nodules had progressed over time and were visible on both legs. The patient had no significant medical history, was a former smoker, used 14 units of alcohol per week, and had done office work. She had not experienced abdominal discomfort or weight loss. Both her parents had died from lung cancer, and there was no further family history of gastrointestinal or pancreatic cancer. She was the mother of three children and used the following medications: allopurinol, metoprolol, omeprazole, perindopril/indapamide, and pravastatin.

**Fig. 1:**
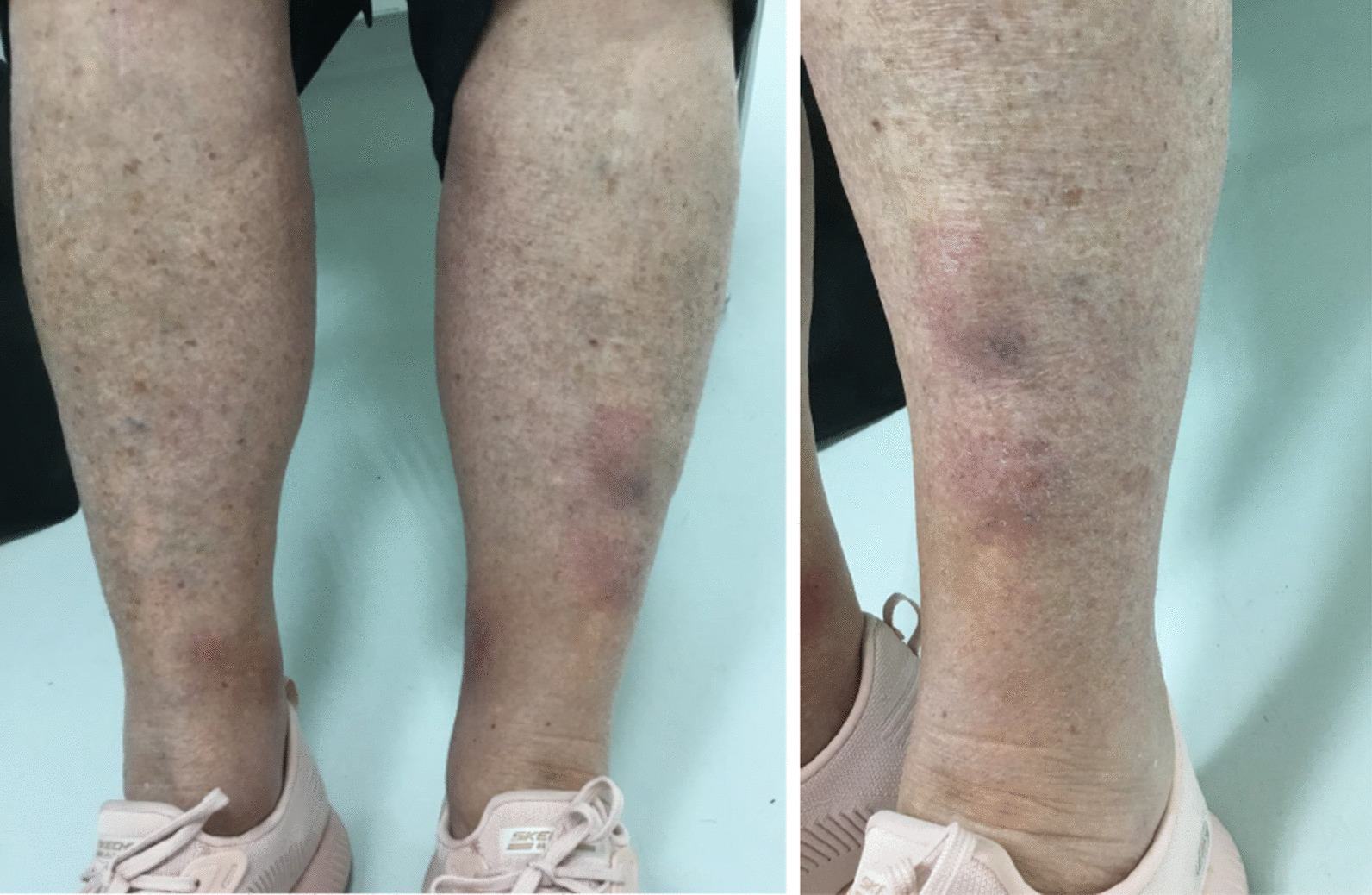
Cutaneous nodules on both legs during presentation

Physical examination revealed three cutaneous nodules ranging in diameter from 1 to 4 cm on both legs (Fig. 1). One was slightly red, while the other two were dark red to purple–blue. All were painful during palpation. According to the dermatologist, the skin lesions were consistent with the clinical diagnosis of panniculitis. Further neurological and physical examination was not performed.

**Fig. 2:**
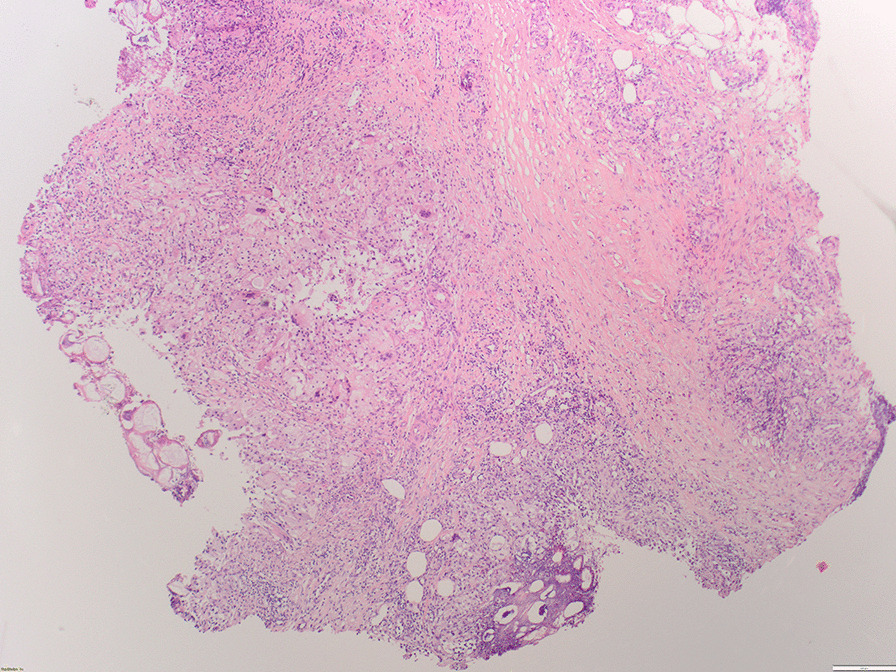
Skin biopsy showing the subcutis with local saponification of fat

**Fig. 3:**
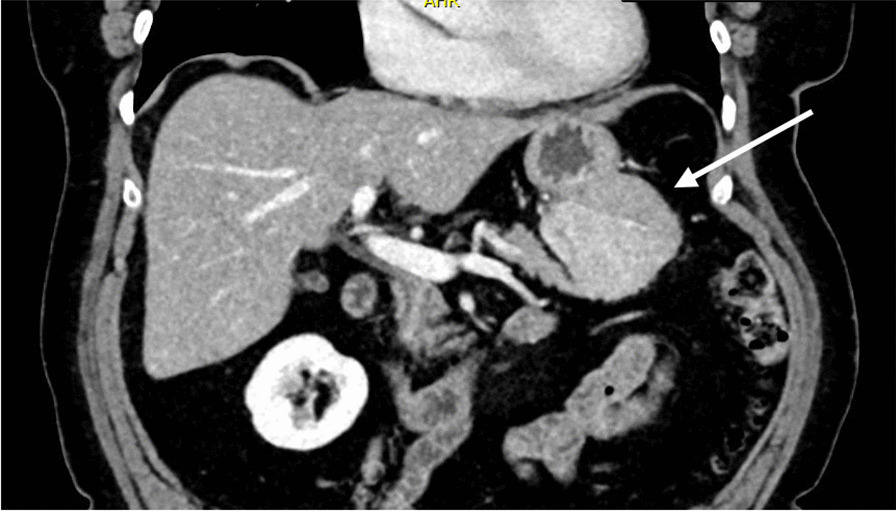
Mass located in the left pancreas, in contact with the stomach

The skin biopsy obtained showed a predominantly lobular panniculitis with fat necrosis of which the aspect was highly suspicious for pancreatic panniculitis (Fig. 2). Based on this finding, further diagnostic analyses were performed including blood tests and computed tomography (CT) scan followed by endoscopic ultrasonography (EUS) and fine-needle biopsy. A lipase serum level of > 3000 U/L (normal range < 60 U/L) and a mass located between the stomach and the left side of the pancreas, with a diameter of 7.4 cm, was seen (Fig. 3). There were no signs of metastases. EUS-guided fine-needle biopsy was obtained, and the histopathological diagnosis was acinar cell carcinoma.

The patient was discussed during the pancreatobiliary multidisciplinary team meeting. Minimally invasive distal pancreatectomy including splenectomy was advised. Upon hospital admission, pulse, blood pressure, and temperature were normal. The patient underwent this procedure laparoscopically including en bloc wedge resection of the stomach owing to tumor in-growth. Operation time was 7 h. The patient was discharged on postoperative day 4 after an uncomplicated hospital stay. During the hospital stay, she received paracetamol, oxycodone, pheneticillin because of the splenectomy, and magnesium hydroxide. Her renal and liver function were normal. No additional urinalysis or microbiology was performed. The cutaneous nodules on both legs had disappeared 1–2 days after surgery.

The diagnosis on the resection specimen was acinar cell carcinoma, the resection margins were free of tumor (R0 resection), and there were no lymph node metastases found in 12 examined lymph nodes. The postoperative multidisciplinary team meeting advised the patient to undergo adjuvant chemotherapy. Nonetheless, the added value of adjuvant chemotherapy for this rare form of pancreatic carcinoma has not been well researched [7–10]. Based on the available data, the patient rejected adjuvant chemotherapy.

During the first and second outpatient visit, at 4 and 6 months postoperatively, no long-term complications were reported, and the patient had recovered well. One year after surgery, the patient presented to the emergency room with similar symptoms as preoperatively. CT scan showed both local recurrence and distal metastases, which were subsequently confirmed by biopsy. She started with palliative folinic acid–fluorouracil–irinotecan–oxaliplatin (FOLFIRINOX) chemotherapy but stopped after two cycles because of disease progression. The patient died 2 months later, 13 months after surgical resection.

## Discussion

We present the case of a 77-year-old woman with no contributing medical history who had cutaneous nodules on her legs as the only symptom of acinar cell carcinoma. The fact that the cutaneous nodules on her legs were the only symptom and that the underlying cause was acinar cell carcinoma makes our case unique. Literature describes pancreatic panniculitis as a rare skin manifestation that appears in approximately 2–3% of all patients with a pancreatic disease [2]. Patients most commonly present with erythematous cutaneous nodules on the lower extremities [4], and up to 45% of patients with pancreatic panniculitis show these nodules before recognition of the underlying pancreatic disease [11]. In addition, acinar cell carcinoma is a very rare pancreatic malignancy that represents only 1–2% of all pancreatic malignancies [8].

For the diagnosis, it is mandatory to obtain a skin biopsy because of the typical histological findings of lobular panniculitis, necrotic anucleate adipocytes called “ghost cells,” and neutrophilic infiltration [12]. Blood tests usually show elevated pancreatic enzymes [13], and instrumental investigations should be performed to diagnose or exclude an underlying pancreatic disease [14].

The prognosis after surgery of resectable pancreatic acinar cell carcinoma is usually more favorable compared with pancreatic ductal adenocarcinoma [9, 15], and moreover a potentially curative treatment [7]. Therefore, surgical intervention is recommended if possible. The added value of adjuvant chemotherapy for this rare form of pancreatic malignancy has not been well researched, and current literature reports varying results on this subject [7–10].

## Conclusion

This case illustrates the importance of clinically recognizing cutaneous nodules as potential warning signs of pancreatic panniculitis due to a (malignant) pancreatic disease. In cases of nonspecific nodules on the lower extremities, a skin biopsy should be obtained alongside routine analysis to diagnose or exclude an underlying pancreatic disease. It is important that the pathologist is aware that certain types of fat necrosis are a sign of a pancreatic disease. A blood test to check for elevated pancreatic enzymes, CT scan of the abdomen, and biopsy are the preferred diagnostic tools to differentiate between a benign or malignant cause. The bottom line is that, in these rare diseases, a multidisciplinary team effort is essential.

## Data Availability

Not applicable.
